# Fatores associados às transições no *status* da violência por parceiro íntimo em pessoas idosas: estudo longitudinal EpiFloripa Idoso

**DOI:** 10.1590/0102-311XPT269525

**Published:** 2026-10-26

**Authors:** Thays Berger Conceição, Deise Warmling, Pierre Guedes de Araujo, Sheila Rubia Lindner

**Affiliations:** 1 Universidade Federal de Santa Catarina, Florianópolis, Brasil.

**Keywords:** Violência por Parceiro Íntimo, Exposição à Violência, Depressão, Abuso de Álcool, Idoso, Intimate Partner Violence, Exposure to Violence, Depression, Alcohol Abuse, Aged, Violencia de Pareja, Exposición a la Violencia, Depresión, Abuso de Alcohol, Anciano

## Abstract

Objetivou-se investigar a associação entre a exposição pregressa à violência, sintomas depressivos e uso de álcool, e as mudanças no status de violência por parceiro íntimo (VPI) em pessoas idosas participantes do estudo EpiFloripa Idoso. É um estudo longitudinal, com dados da coorte analisados por regressão logística multinomial. O desfecho VPI foi categorizado segundo a mudança no status da violência ao longo do tempo, para perpetração e vitimização. A amostra final foi de 336 participantes, aqueles que responderam ao instrumento nas duas medidas de tempo. Como resultado, os fatores associados ao início da perpetração foram: sintomas depressivos com histórico de violência (RC = 4,93; IC95%: 1,10-21,30) e consumo elevado de álcool (RC = 2,77; IC95%: 1,12-6,84). A continuação da perpetração foi associada a sintomas depressivos com (RC = 8,09; IC95%: 2,34-27,97) e sem (RC = 4,44; IC95%: 1,18-16,60) histórico de violência. O início da vitimização foi associado com sintomas depressivos com histórico de violência (RC = 9,40; IC95%: 1,94-45,47). Já a continuidade da vitimização foi associada a sintomas depressivos com (RC = 10,20; IC95%: 2,48-41,94) e sem histórico de violência (RC = 8,04; IC95%: 1,91-33,81). Sugere-se a necessidade de ações integradas em saúde mental e de enfrentamento à violência em crianças e pessoas idosas, que podem potencialmente auxiliar a prevenir a VPI futura. Entre as limitações, destacam-se o uso de medidas autorreferidas, potencial viés de memória, limitações do instrumento para a população idosa e restrições analíticas decorrentes do tamanho amostral.

## Abstract (en)

This study aimed to investigate the association between previous exposure to violence, depressive symptoms, and alcohol use and changes in intimate partner violence status in older adults in EpiFloripa Idoso. This longitudinal study analyzed cohort data by multinomial logistic regression. It categorized intimate partner violence according to violence (perpetration and victimization) status change over time. Its final sample consisted of 336 participants (those who answered the instrument in both time measurements). The following factors were associated with the onset of the perpetration: depressive symptoms with a history of violence (OR = 4.93; 95%CI: 1.10-21.30) and high alcohol consumption (OR = 2.77; 95%CI: 1.12-6.84). Perpetration continuity was associated with depressive symptoms with (OR = 8.09; 95%CI: 2.34-27.97) and without (OR = 4.44; 95%CI: 1.18-16.60) history of violence. The onset of victimization was associated with depressive symptoms with a history of violence (OR = 9.40; 95%CI: 1.94-45.47). Victimization continuity was associated with depressive symptoms with (OR = 10.20; 95%CI: 2.48-41.94) and without history of violence (OR = 8.04; 95%CI: 1.91-33.81). This study finds the need for integrated actions in mental health and in coping with violence in children and older adults as they can help to prevent future intimate partner violence. Limitations include self-reported measures, potential recall bias, limitations of the instrument for older adults, and analytical restrictions due to the sample size.

## Abstract (es)

El objetivo fue investigar la asociación entre la exposición previa a la violencia, los síntomas depresivos y el consumo de alcohol, y los cambios en el estatus de violencia de pareja íntima (VPI) en adultos mayores que participan en el estudio EpiFloripa Idoso. Se trata de un estudio longitudinal, con datos de cohorte analizados mediante regresión logística multinomial. El resultado del VPI se clasificó según los cambios en el estatus de violencia a lo largo del tiempo, tanto para los perpetradores como para las víctimas. La muestra final estuvo compuesta por 336 participantes, aquellos que respondieron al instrumento en ambas mediciones de tiempo. Como resultado, los factores asociados con el inicio de la perpetración fueron: síntomas depresivos con antecedentes de violencia (RC = 4,93; IC95%: 1,10-21,30) y alto consumo de alcohol (RC = 2,77; IC95%: 1,12-6,84). La perpetración continuada se asoció con síntomas depresivos con (RC = 8,09; IC95%: 2,34-27,97) y sin (RC = 4,44; IC95%: 1,18-16,60) antecedentes de violencia. El inicio de la victimización se asoció con síntomas depresivos con antecedentes de violencia (RC = 9,40; IC95%: 1,94-45,47). A su vez, la victimización continua se asoció con síntomas depresivos con (RC = 10,20; IC95%: 2,48-41,94) y sin antecedentes de violencia (RC = 8,04; IC95%: 1,91-33,81). Se sugiere que son necesarias iniciativas integradas en materia de salud mental y de enfrentamiento de la violencia contra los niños y adultos mayores, lo que podría ayudar a prevenir futuros casos de VPI. Entre las limitaciones, destacan el uso de medidas autoinformadas, el posible sesgo de memoria, las limitaciones del instrumento para población anciana y las restricciones analíticas derivadas del tamaño de la muestra.

## Introdução

A violência por parceiro íntimo (VPI) é descrita pela Organização Mundial da Saúde (OMS) como aquela que ocorre dentro de uma relação conjugal, abrangendo qualquer comportamento, incluindo os atos de agressão física, psicológica, sexual, comportamentos controladores, violência financeira, entre outros, que possam resultar em prejuízos à saúde ^1^. Para mensurar as taxas desse fenômeno, existe um grande desafio a ser superado, pois as pesquisas acompanham a sociedade no que se refere ao preconceito institucional e social em relação à idade, excluindo, na maioria das vezes, as pessoas idosas dos estudos, ao adotar como ponto de corte a idade reprodutiva ^2^ ^,^ ^3^. Contribuindo para a dificuldade em se obter estimativas dessa população, frequentemente os estudos não distinguem a população idosa dos adultos nas etapas de análise de dados, nem na descrição dos resultados ^4^. Além disso, nos estudos que investigam o abuso de idosos, é comum que a VPI seja agrupada com outras formas de violência praticadas por cuidadores, filhos e outras pessoas próximas ^5^, generalizando situações multidimensionais em uma única categoria.

Apesar das limitações metodológicas, evidências destacam a ocorrência desse fenômeno nessa população. Uma revisão sistemática sugere que a prevalência global de VPI varia entre 1,8% e 5,9% para a violência física, 1,2% para a sexual e entre 1,9% e 36,1% para a psicológica, em amostras exclusivamente com pessoas idosas ^6^. Estudos internacionais e nacionais corroboram a magnitude do problema. No México, estima-se que entre 8,1% e 18,6% das pessoas idosas são vítimas de algum tipo de violência, sendo que 20,3% desses casos são cometidos pelo atual companheiro ^7^. No Brasil, dados da segunda onda do estudo *EpiFloripa: Condições de Saúde de Idosos de Florianópolis* (EpiFloripa Idoso), realizado em Florianópolis, Santa Catarina, revelam que, entre os 651 participantes acompanhados, 48,3% dos homens e 46,4% das mulheres relataram ter sofrido VPI no último ano ^8^.

A complexidade da VPI em pessoas idosas também reside em suas múltiplas trajetórias de manifestação. A literatura sugere que essa forma de violência pode ocorrer como uma continuidade de episódios iniciados na vida adulta ou surgir de maneira inédita na fase idosa da vida, em um relacionamento longo já existente ou em um novo relacionamento ^9^ ^,^ ^10^. Compreendê-la como um processo cumulativo, ao invés de eventos isolados, permite reconhecer que os padrões de violência podem variar ao longo dos anos ^11^ ^,^ ^12^, e são profundamente afetados pelas normas sociais que estruturam as relações conjugais ao longo da vida, como a desigualdade de gênero.

Pessoas idosas foram socializadas em padrões de gênero marcados por relações de poder desiguais, existiam expectativas de privacidade em assuntos familiares, a subserviência das esposas, a aceitabilidade da violência para manter a ordem doméstica, o estigma do divórcio e a dependência econômica ^13^ ^,^ ^14^. No Brasil, foi apenas em 1962, com a *Lei nº 4.121* ^15^, que ocorreu a modificação sobre a condição jurídica da mulher casada, concedendo direito de viajar, abrir conta em banco, ter a guarda dos filhos, trabalhar, além de abolir mais algumas proibições.

Alguns estudos indicam que o avanço da idade está associado a uma redução na taxa de violência física e sexual ^6^ ^,^ ^16^, sendo que pessoas idosas mais jovens tendem a relatar com maior frequência situações de violência íntima ^17^. No entanto, não é possível prever quais indivíduos apresentarão diminuição e quais manterão comportamentos agressivos ao longo do tempo. À medida que a expectativa de vida aumenta, amplia a possibilidade de se envolver em novos relacionamentos violentos ou de permanecer em uma relação abusiva por mais tempo, potencializando as consequências da violência, especialmente no contexto do envelhecimento ^3^.

A VPI em pessoas idosas traz graves consequências para a saúde física e mental e é responsável por parte das hospitalizações, institucionalização e mortalidade ^18^, além de afetar de forma significativa o bem-estar psicológico dessa população. Em uma coorte de mulheres australianas com idades entre 70 e 75 anos, o histórico de vitimização pelo parceiro esteve associado significativamente a maiores chances de relato de pior bem-estar psicológico e de depressão durante o período de acompanhamento ^19^. Outro estudo identificou que a vitimização por VPI tem maior probabilidade de ocorrer naquelas que usam medicamentos para dormir, para se acalmar ou para depressão em pessoas com 60 anos ou mais ^17^.

Uma pesquisa que compara adultos e pessoas idosas envolvidos em VPI evidencia que, independentemente da faixa etária, ter uma deficiência ou um problema de saúde físico, mental ou de outro tipo de longa duração aumenta a probabilidade de denúncia de abuso emocional/financeiro e físico/sexual ^20^. Ainda, os autores ^20^ destacam que ter um parceiro que consome mais de cinco doses de bebida alcoólica em uma única ocasião está associado a todas as formas de violência, com a chance mais elevada entre as pessoas idosas (razão de chances - RC = 4,14; intervalo de 95% de confiança - IC95%: 2,18-7,86) do que nos adultos (RC = 3,35; IC95%: 2,39-4,69). Esses achados são corroborados por Poole & Rietschlin ^17^, que observaram, em uma amostra de pessoas com 60 anos ou mais, a associação entre a frequência do consumo de álcool pelo parceiro (de duas a três vezes por semana ou episódios de consumo excessivo) e um aumento do risco de abuso financeiro (0,5%) e físico (1,3%). Na Tanzânia, em amostra com adultos e pessoas idosas, a ocorrência de VPI sexual esteve associada ao consumo de álcool por parte dos homens e à saúde mental fragilizada, e a experiência de violência na infância foi associada às mulheres ^21^.

A relação entre sintomas depressivos, consumo de álcool e VPI é complexa e potencialmente bidirecional. Evidências indicam que tanto o uso problemático de álcool ^22^ quanto sintomas depressivos ^23^ ^,^ ^24^ podem atuar como antecedentes e consequências da violência, além de, em alguns contextos, funcionar como possíveis mediadores. Essa inter-relação sugere que diferentes mecanismos podem estar envolvidos nessa dinâmica ao longo do curso da vida. Nesse contexto, as vulnerabilidades prévias podem contribuir para riscos mais altos.

Destaca-se também o papel da exposição à violência familiar na infância como um determinante de longo prazo da VPI. Como visto em membros da comunidade chinesa nos Estados Unidos com idade igual ou superior a 60 anos, a chance foi aumentada para VPI psicológica (RC = 7,60; IC95%: 4,29-13,45) e física/sexual (RC = 4,06; IC95%: 1,71-9,63) ^25^. Corroborando esses dados, Brassard et al. ^26^ indicam que todas as formas de maus-tratos infantis pesquisadas - negligência, abuso físico, abuso psicológico, testemunhar a violência parental e abuso sexual - previram um risco aumentado de vitimização por VPI em mulheres adultas e idosas do Canadá.

A perspectiva de que a violência acompanha as pessoas ao longo do curso da vida tem sido discutida. Diferentes teorias buscam explicar os mecanismos pelos quais experiências de violência na infância influenciam a violência perpetrada ou sofrida nos relacionamentos íntimos posteriores. O modelo Vulnerabilidade-Estresse-Adaptação propõe que a violência emerge da interação entre três tipos de processos: vulnerabilidade duradoura, estressores e processo de adaptação do casal, e as experiências adversas, como sofrer violência e a exposição à violência doméstica, são vulnerabilidades duradouras que constituem e podem aumentar o risco de VPI ao longo da vida ^26^. Outra forma de olhar para essa questão é pela abordagem explicativa da teoria da aprendizagem social, proposta por Bandura ^27^, segundo a qual comportamentos violentos podem ser aprendidos por meio da observação e modelagem de interações familiares, sendo posteriormente reforçados por mecanismos simbólicos e sociais, como pares, cultura, instituições etc. Em ambas as explicações a exposição não assume uma relação causal linear com a VPI.

Evidências empíricas mostraram que experiências traumáticas na infância, como a exposição à violência familiar, têm efeitos duradouros, afetando a saúde mental ^28^ ^,^ ^29^, os comportamentos de saúde, como o uso de álcool ^30^ e as relações interpessoais das pessoas idosas ^31^. Porém, os efeitos temporais desses traumas no funcionamento posterior da vida foram pouco explorados ^32^. Além disso, há uma necessidade urgente de estimativas atuais de VPI em populações idosas, tanto globalmente quanto em regiões específicas ^33^. Dada a complexidade do tema, torna-se essencial considerar a intersecção entre vitimização e perpetração da violência, depressão, uso de álcool e o histórico de violência familiar infantil. Diante desse contexto, este estudo busca investigar a associação entre a exposição pregressa à violência, sintomas depressivos e o uso de álcool, e as mudanças no *status* de VPI em pessoas idosas participantes do estudo EpiFloripa Idoso.

## Método

Esta pesquisa de base populacional é oriunda das informações da segunda (2013-2014) e terceira (2017-2019) ondas do inquérito EpiFloripa Idoso, que buscou investigar as condições de saúde de homens e mulheres com 60 anos ou mais completados até o ano de início da pesquisa, moradores da zona urbana de Florianópolis.

A linha de base ocorreu em 2009-2010. O tamanho da amostra foi calculado com base em uma população de 44.460 habitantes idosos, com nível de 95% de confiança e prevalência de 50% para manifestação desconhecida. Além disso, adotou-se um erro amostral de quatro pontos percentuais e um efeito de delineamento amostral estimado em dois. Para compensar possíveis perdas, foram acrescidos 20% ao total, juntamente com 15% adicionais para análises de associação, resultando em uma amostra final de 1.599 participantes ^34^.

A obtenção dos dados ocorreu por meio de entrevistas presenciais conduzidas por entrevistadores treinados. A qualidade das informações foi garantida pela checagem semanal e verificação que reproduzia aleatoriamente, em aproximadamente 10% dos participantes, uma mini entrevista via telefone. A amostra deste estudo foi composta por participantes da pesquisa EpiFloripa Idoso que relataram possuir parceiro nos últimos 12 meses e responderam ao *Conflict Tactics Scales Form* [*Escala de Táticas de Conflito*] (CTS-1, acrônimo em inglês) na segunda e terceira ondas da pesquisa.

O desfecho foi a VPI, tanto sofrida como perpetrada. Utilizou-se o instrumento CTS-1 traduzido e adaptado para o português do Brasil ^35^. O CTS-1 investiga três formas de manejo de conflitos: (i) argumentação racional e moderada, medida por dois itens; (ii) violência psicológica, analisada por seis itens que envolvem insultos e ameaças; e (iii) violência física, avaliada por nove itens relacionados ao uso de força física ou agressões explícitas. O entrevistado responde sobre os atos praticados tanto por ele (violência perpetrada) quanto pelo parceiro (violência sofrida), com respostas “sim” ou “não”. Considerou-se a presença de VPI quando houve pelo menos uma resposta positiva em qualquer item das subescalas de violência psicológica ou física. Para a análise estratificada do desfecho, a VPI perpetrada foi categorizada em quatro grupos segundo a mudança no *status* da violência com o tempo: (1) permaneceu sem perpetrar, (2) passou a perpetrar, (3) deixou de perpetrar e (4) continuou perpetrando. O mesmo foi feito com a VPI sofrida: (1) permaneceu sem sofrer, (2) passou a sofrer, (3) deixou de sofrer e (4) continuou sofrendo.

As variáveis exploratórias sociodemográficas foram: sexo (homens, mulheres); idade (60-69, 70-79, ≥ 80 anos); escolaridade em anos de estudo (sem escolaridade formal, 1-4, 5-8, 9-11, ≥ 12); renda familiar mensal em salários mínimos (até 1, entre 1 e 5, entre 5 e 10, > 10).

As variáveis de exposição relacionadas à saúde foram o consumo de álcool e a suspeita de depressão. A frequência do consumo de bebidas alcoólicas foi categorizada com base nas três primeiras questões do *Alcohol Use Disorders Identification Test* [Teste de Identificação de Distúrbios por Uso de Álcool] (AUDIT, acrônimo em inglês) ^36^, em três categorias: nunca - indivíduos que referiram não consumir bebidas alcoólicas; moderado - consumo de até duas doses de álcool em um dia comum, sem relatos de consumo de cinco ou mais doses em uma única ocasião; e alto - consumo de mais de duas doses em um dia normal ou de cinco ou mais doses em uma única ocasião.

A suspeita de depressão foi identificada por meio da versão reduzida do *Geriatric Depression Scale* [*Escala de Depressão Geriátrica*] (GDS, acrônimo em inglês), validada no Brasil, composta por 15 questões ^37^. Instrumento para detectar episódios depressivos maiores, seguindo os critérios da 10ª revisão da Classificação Internacional de Doenças (CID-10) e do Manual Diagnóstico e Estatístico de Transtornos Mentais (DSM-IV, acrônimo em inglês) ^37^. Os sintomas foram autorreferidos e o ponto de corte foi de cinco para ausência de sintomas depressivos e seis ou mais para presença de sintomas de depressão.

A exposição à violência na infância foi medida com base em três perguntas: “Você viu ou ouviu seus pais batendo uns nos outros?”; “Você foi espancado por seus pais ou outros membros da sua família?”; “Você sofreu abuso sexual de algum familiar durante a infância?”. As três variáveis foram analisadas de forma independente e posteriormente categorizadas de forma dicotômica (sem histórico de violência/com histórico de violência). Detalhes adicionais sobre o estudo EpiFloripa Idoso estão descritos na publicação de D’Orsi et al. ^38^.

Para a caracterização da amostra, realizou-se estatística descritiva dos participantes da segunda e terceira ondas, apresentando-se frequências absolutas e relativas acompanhadas dos respectivos IC95%. Com o objetivo de identificar os fatores associados às mudanças no *status* da VPI perpetrada e sofrida entre as duas ondas nas pessoas idosas participantes do estudo EpiFloripa Idoso, usou-se a análise de regressão logística multinomial bruta e ajustada, com estimativas de RC e seus respectivos IC95%.

Para a análise multinomial, os desfechos de VPI perpetrada e sofrida foram estratificados segundo o *status* de mudança. A variável “sintomas depressivos” foi agrupada com violência pregressa e dividida em quatro categorias: “ausência de sintomas depressivos sem histórico de violência”, “ausência de sintomas depressivos com histórico de violência”, “presença de sintomas depressivos sem histórico de violência” e “presença de sintomas depressivos com histórico de violência”. Essa divisão apresenta sustentação teórica na literatura ^32^ ^,^ ^39^ ^,^ ^40^ ^,^ ^41^. Foi testado o modelo nulo e, após, testado o modelo ajustado para as variáveis de controle (idade, anos de escolaridade e renda). As categorias de referência das análises foram “permaneceu sem perpetrar VPI” e “permaneceu sem sofrer VPI”. Foram realizadas as análises no software estatístico Stata, versão 14.0 (https://www.stata.com). Consideraram-se o efeito de desenho e os pesos amostrais em todas as análises feitas neste estudo, conforme detalhado em Schneider et al. ^42^.

A pesquisa recebeu a aprovação do Comitê de Ética em Pesquisa com Seres Humanos da Universidade Federal de Santa Catarina. Os números de aprovação foram 352/2008 para a linha de base, 329.650 para a segunda onda e 1.957.977 para a terceira, sob o Certificado de Apresentação para Apreciação Ética (CAAE) 16731313.0.0000.0121. 

## Resultados

Das 1.197 pessoas idosas entrevistadas no início da segunda onda do estudo, 137 morreram, 178 se recusaram e 139 foram consideradas perdas, resultando em 743 entrevistados para a terceira onda da pesquisa. Os demais participantes da amostra total - provenientes do estudo EpiFloripa Adulto ou novos integrantes que ingressaram em reposição das perdas amostrais - não foram incluídos nesta subamostra por não terem respondido ao questionário CTS-1 na segunda onda. Dos 743 participantes em acompanhamento, 351 responderam ao questionário CTS-1 na terceira onda. Outros 15 foram excluídos da análise por não terem respondido ao CTS-1 na segunda onda. A amostra final correspondeu a 25,2% dos participantes entrevistados na terceira onda do estudo (2017-2019) (Figura 1).

**Figura 1:**
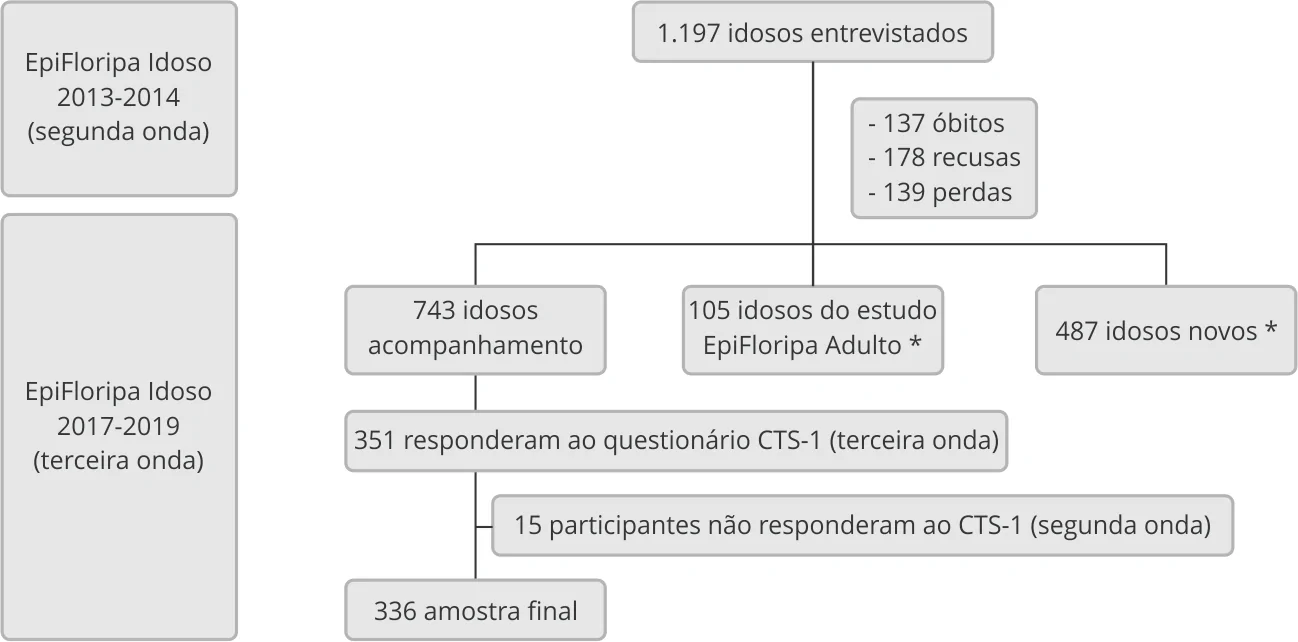
Fluxograma do tamanho da amostra do estudo segundo distribuição do número de participantes, perdas e recusas do estudo de coorte EpiFloripa Idoso. Florianópolis, Santa Catarina, Brasil, 2013-2014 e 2017-2019.

CTS-1: *Conflict Tactics Scales Form*. Fonte: elaboração própria. * Participantes da terceira onda, mas não elegíveis para o presente estudo.

Dentre os participantes, predominaram nas duas ondas analisadas pessoas na faixa etária de 70-79 anos (59,4% e 58,9%), que relataram não consumir bebidas alcoólicas (47,2% e 45%) e sem sintomas depressivos (88% e 86,3%). Não foram encontradas diferenças estatisticamente significativas entre as categorias das demais variáveis analisadas, bem como entre as amostras das duas ondas analisadas (Tabela 1).

**Tabela 1:** Características sociodemográficas, condições de saúde e comportamentais dos participantes do estudo de coorte EpiFloripa Idoso que responderam ao *Conflict Tactics Scales Form* (CTS-1). Florianópolis, Santa Catarina, Brasil, 2013-2014 e 2017-2019.

Variáveis	2013-2014 (n = 382)		2017-2019 (n = 351)	
	n	% (IC95%)	n	% (IC95%)
Sexo				
Feminino	199	49,2 (43,4-54,8)	172	47,6 (40,9-53,9)
Masculino	183	50,8 (45,1-56,5)	179	52,5 (46,0-59,0)
Faixa etária (anos)				
60-69	66	16,6 (11,3-23,6)	68	19,1 (13,5-23,8)
70-79	222	59,4 (53,1-65,4)	204	58,9 (52,8-63,1)
≥ 80	94	24,0 (18,0-31,0)	79	21,8 (15,7-27,1)
Escolaridade (anos de estudo)				
Sem escolaridade formal	19	3,6 (1,9-6,6)	17	3,3 (1,8-5,9)
1-4	111	25,6 (18,8-33,9)	102	25,1 (18,5-33,4)
5-8	77	19,1 (13,9-25,6)	65	18,7 (13,4-22,9)
9-11	61	18,6 (13,5-25,1)	56	18,8 (13,6-25,5)
≥ 12	114	32,9 (26,2-40,4)	111	33,7 (26,6-36,6)
Renda familiar (salários mínimos)				
Até 1	24	5,4 (2,9-9,7)	20	5,3 (2,8-9,6)
Entre 1 e 5	184	42,2 (33,3-51,6)	162	39,4 (30,4-49,4)
Entre 5 e 10	90	25,7 (19,3-33,2)	86	26,7 (19,9-34,8)
> 10	84	26,6 (19,9-34,6)	83	28,4 (21,2-36,3)
Consumo de álcool [p = 0,591]				
Nunca	196	47,2 (38,9-55,6)	173	45,0 (36,6-53,8)
Moderado	108	27,6 (22,2-33,9)	103	28,4 (22,8-34,9)
Alto	78	25,2 (17,2-35,0)	75	26,4 (18,0-36,9)
Sintomas depressivos				
Ausência	328	88,0 (82,1-92,1)	303	86,3 (82,3-89,5)
Presença	54	12,0 (7,8-17,8)	48	13,7 (10,4-17,6)
Presenciou violência entre os pais na infância *				
Não	-	-	313	89,0 (82,8-93,1)
Sim	-	-	38	11,0 (6,8-17,1)
Apanhou de algum familiar na infância *				
Não	-	-	159	47,2 (39,1-54,5)
Sim	-	-	192	52,8 (44,4-60,8)
Sofreu abuso sexual de algum familiar na infância *				
Não	-	-	347	98,6 (94,2-99,6)
Sim	-	-	4	1,4 (0,3 - 5,7)
Violência pregressa *				
Sem histórico de violência	-	-	149	44,4 (37,0-52,1)
Com histórico de violência	-	-	202	55,6 (47,8-62,9)

IC95%: intervalo de 95% de confiança.

Fonte: elaboração própria.

* As questões sobre histórico de violência pregressa foram incluídas na terceira onda do estudo.

Em relação às mudanças no *status* de violência perpetrada ao longo das duas ondas do estudo, a maioria das mulheres (73,51%) e dos homens (66,50%) manteve o comportamento relativo à perpetração da VPI ao longo do seguimento. A proporção de mulheres que permaneceram sem perpetrar (45,08%) foi numericamente superior às que continuaram a perpetrar a VPI (28,43%), diferentemente para homens (31,03% *vs*. 35,47%). Observou-se a predominância, com significância estatística, de pessoas idosas que permaneceram sem perpetrar VPI cuja idade era maior do que 80 anos (48,70%; IC95%: 32,82-64,81) e que não apanharam na infância (43,34%; IC95%: 34,41-52,73). Nas demais variáveis sociodemográficas, de saúde e comportamentais analisadas, não foram verificadas diferenças significativas estatisticamente (Tabela 2).

**Tabela 2:** Mudança no *status* da violência perpetrada em idosos do estudo de coorte EpiFloripa Idoso de acordo com as variáveis sociodemográficas, de saúde e comportamentais. Florianópolis, Santa Catarina, Brasil, 2013-2014 e 2017-2019.

Variáveis	Permaneceu sem perpetrar		Passou a perpetrar		Deixou de perpetrar		Continuou perpetrando	
	n	% (IC95%)	n	% (IC95%)	n	% (IC95%)	n	% (IC95%)
Total (N = 336)	119	38,00 (29,32-47,35)	55	14,19 (10,09-19,59)	53	15,63 (11,18-21,42)	109	32,16 (24,88-40,42)
Sexo								
Feminino	65	45,08 (33,97-58,28)	22	12,11 (6,51-21,43)	24	13,55 (8,28-21,40)	53	28,43 (20,09-38,56)
Masculino	54	31,03 (20,55-43,88)	33	16,02 (10,09-24,48)	29	17,47 (11,48-25,67)	56	35,47(25,83-46,44)
Faixa etária (anos)								
60-69	18	35,06 (16,01-60,45)	9	11,96 (5,08-25,63)	10	17,47 (7,49-35,63)	25	35,50 (17,48-58,84)
70-79	70	34,95 (25,52-45,73)	33	12,77 (7,79-20,24)	28	14,92 (9,77-22,13)	68	37,34 (27,99-47,74)
≥ 80	31	48,70 (32,82-64,81)	13	19,86 (10,65-33,98)	15	16,05 (7,74-30,33)	16	15,38 (7,11-30,12)
Escolaridade (anos de estudo)								
Sem escolaridade formal	5	35,92 (13,53-66,76)	4	18,91 (7,55-39,97)	4	31,11 (12,53-57,73)	3	14,04 (4,17-37,98)
1-4	30	34,99 (22,67-49,70)	15	11,55 (6,57-19,52)	19	21,68 (13,20-33,49)	31	31,76 (19,11-47,82)
5-8	22	29,53 (17,16-45,86)	11	18,67 (8,00-37,72)	8	13,12 (5,52-28,05)	22	38,67 (25,26-54,05)
9-11	23	40,78 (25,20-58,46)	7	10,38 (4,24-23,26)	8	16,41 (7,60-31,89)	17	32,41 (16,02-54,65)
≥ 12	39	43,38 (29,84-57,99)	18	15,39 (8,43-26,42)	14	10,68 (4,39-21,58)	36	30,53 (19,61-44,19)
Renda familiar (salários mínimos)								
Até 1	5	17,09 (5,83-40,71)	2	5,39 (11,36-22,05)	6	39,02 (15,41-69,20)	6	38,48 (12,13-73,90)
Entre 1 e 5	49	31,96 (21,51-44,59)	23	12,29 (8,07-18,26)	26	17,43 (10,87-26,76)	58	38,48 (12,13-73,90)
Entre 5 e 10	34	47,98 (33,36-62,96)	19	13,64 (7,27-24,15)	8	10,50 (4,41-22,98)	19	38,31 (28,52-49,14)
> 10	31	41,00 (28,14-55,22)	11	18,84 (9,26 -34,57)	13	13,64 (6,77-25,50)	26	26,50 (14,49-43,40)
Consumo de álcool								
Nunca	61	42,88 (29,91-56,90)	21	11,11 (6,60-18,18)	29	15,55 (9,41-24,61)	54	30,41 (19,98-43,35)
Moderado	38	33,92 (21,76-48,65)	19	22,48 (11,30-29,75)	15	19,48 (10,60-33,05)	26	24,10 (15,58-35,34)
Alto	20	34,16 (17,68-55,60)	15	10,54 (4,87-21,33)	9	11,68 (5,95-21,68)	29	43,60 (23,59-65,93)
Sintomas depressivos								
Ausência	111	40,96 (31,48-51,16)	47	14,34 (9,87-20,39)	46	15,31 (10,82-21,21)	87	29,37 (21,98-38,03)
Presença	8	13,54 (5,85 - 28,30)	8	12,90 (5,21-28,49)	7	18,29 (6,54-41,72)	22	55,25 (31,75-76,61)
Presenciou violência entre os pais na infância								
Não	113	40,54 (31,71-50,04)	50	14,95 (10,37-21,06)	48	16,12 (11,60-21,95)	88	28,38 (21,54-36,37)
Sim	6	17,80 (5,42-44,98)	5	8,14 (2,65-22,34)	5	11,75 (3,99-29,93)	21	62,29 (34,41-80,75)
Apanhou de algum familiar na infância								
Não	61	43,34 (34,41-52,73)	20	9,09 (5,13-15,61)	33	22,29 (15,30-31,30)	38	25,25 (18,13-34,01)
Sim	58	33,25 (23,27-45,00)	35	18,72 (11,77-28,45)	20	9,70 (5,72-15,96)	71	38,31 (27,58-50,31)
Sofreu abuso sexual de algum familiar na infância								
Não	117	38,10 (29,37-47,68)	55	14,39 (10,23-19,86)	52	15,36 (10,90-21,20)	108	32,13 (24,79-40,47)
Sim	2	31,02 (3,36-85,30)	0	-	1	34,48 (13,55-63,86)	1	34,48 (13,55-63,86)
Violência pregressa								
Sem histórico de violência	50	45,34 (36,44-54,54)	20	9,69 (5,36-16,90)	29	21,38 (14,20-30,88)	34	23,57 (16,56-32,40)
Com histórico de violência	60	32,20 (22,47-43,76)	35	17,75 (11,12-27,12)	24	11,07 (6,59-18,02)	75	38,96 (28,19-50,92)

IC95%: intervalo de 95% de confiança.

Fonte: elaboração própria.

Quanto às mudanças do *status* da violência sofrida no período de acompanhamento, constatou-se que a maioria dos participantes (68,10%) manteve o *status* relativo à vitimização por VPI. Desses, a proporção das pessoas idosas sem sofrer violência (36,74%) foi ligeiramente superior em relação aos que continuaram a sofrer (31,36%). Não houve diferenças estatísticas significativas entre as variáveis sociodemográficas, de saúde e comportamentais analisadas e as mudanças no *status* de VPI sofridas (Tabela 3).

**Tabela 3:** Mudança no *status* de violência sofrida em idosos do estudo de coorte EpiFloripa Idoso de acordo com as variáveis sociodemográficas, de saúde e comportamentais. Florianópolis, Santa Catarina, Brasil, 2013-2014 e 2017-2019.

Variáveis	Permaneceu sem sofrer		Passou a sofrer		Deixou de sofrer		Continuou sofrendo	
	n	% (IC95%)	n	% (IC95%)	n	% (IC95%)	n	% (IC95%)
Total (N = 336)	125	36,74 (28,08-46,36)	46	13,19 (9,15-18,62)	61	18,69 (14,18-24,22)	104	31,36 (23,08-41,02)
Sexo								
Feminino	63	38,39 (28,63-49,19)	22	14,42 (8,19-24,14)	27	18,49 (11,66-28,05)	51	28,68 (20,09-39,16)
Masculino	62	35,31 (25,26-46,86)	24	12,13 (7,49-19,06)	34	18,86 (12,95-26,63)	53	33,68 (21,76-48,11)
Faixa etária (anos)								
60-69	21	38,28 (18,21-63,33)	5	9,14 (3,19-23,50)	15	19,29 (8,85-37,06)	21	33,26 (15,79-56,98)
70-79	73	36,55 (26,79-47,56)	32	15,85 (9,78-24,66)	31	15,27 (9,72-23,16)	63	32,31 (23,27-42,90)
≥ 80	31	36,01 (19,87-56,09)	9	9,15 (4,04-19,39)	15	27,64 (15,29-44,71)	20	27,18 (14,63-44,83)
Escolaridade (anos de estudo)								
Sem escolaridade formal	5	35,92 (13,53-66,76)	2	8,68 (2,20-28,61)	6	41,34 (15,91-72,40)	3	14,04 (4,17-37,98)
1-4	35	36,61 (22,31-53,74)	13	13,95 (7,81-23,70)	20	22,78 (13,67-35,45)	28	26,63 (14,05-44,64)
5-8	23	32,98 (19,62-49,82)	10	21,47 (9,38-41,93)	11	12,47 (5,87-24,56)	20	33,05 (21,20-47,52)
9-11	23	42,64 (27,95-58,75)	4	5,37 (1,65-16,08)	6	15,29 (6,51-31,51)	21	36,69 (19,06-58,78)
≥ 12	39	35,66 (22,04-52,08)	17	12,90 (7,24-21,93)	18	18,73 (10,05-32,21)	32	32,69 (20,92-47,12)
Renda familiar (salários mínimos)								
Até 1	9	37,08 (14,24-67,65)	1	2,10 (0,0-14,72)	5	27,01 (8,64-59,16)	4	33,79 (0,08-73,16)
Entre 1 e 5	52	31,52 (22,20-42,61)	18	11,01 (0,6-19,35)	30	20,86 (14,41-29,22)	56	36,59 (25,49-49,32)
Entre 5 e 10	35	49,20 (35,75-61,76)	14	12,99 (6,12-25,45)	10	11,07 (4,90-23,13)	21	26,73 (13,90-45,17)
> 10	29	32,76 (19,19-49,98)	13	18,40 (9,42-32,83)	16	21,04 (11,13-36,18)	23	27,78 (16,01-43,70)
Consumo de álcool								
Nunca	63	40,48 (27,88-54,48)	20	11,99 (7,11-19,51)	35	20,11 (12,01-31,71)	47	27,40 (17,04-40,94)
Moderado	39	32,57 (21,59-45,86)	13	13,60 (6,16-27,40)	16	21,60 (11,67-36,49)	30	32,22 (20,50-46,70)
Alto	23	34,76 (18,29-55,92)	13	14,82 (6,82-29,24)	10	13,17 (7,27-22,70)	27	37,23 (19,76-58,82)
Sintomas depressivos								
Ausência	119	39,64 (30,12-50,02)	38	13,32 (9,01-19,25)	51	18,36 (13,77-24,05)	84	28,66 (20,09-39,10)
Presença	6	12,12 (48,38-27,25)	8	12,13 (0,4-27,27)	10	21,49 (0,08-44,30)	20	54,24 (30,96-75,80)
Presenciou violência entre os pais na infância								
Não	117	39,50 (29,57-50,39)	40	13,37 (9,07-19,28)	55	18,78 (13,89-24,90)	87	28,33 (20,24-38,10)
Sim	8	14,52 (0,06-31,03)	6	11,77 (3,08-35,83)	6	17,92 (5,57-44,69)	17	55,77 (31,91-77,24)
Apanhou de algum familiar na infância								
Não	62	40,14 (30,03-51,17)	17	9,89 (5,46-17,27)	35	26,19 (18,96-34,97)	38	23,76 (16,94-32,26)
Sim	63	33,76 (24,06-45,06)	29	16,08 (9,88-25,10)	26	12,12 (7,46-19,07)	66	38,01 (26,19-51,45)
Violência pregressa								
Sem histórico de violência	58	40,65 (30,12-52,12)	17	10,54 (5,77-18,48)	33	26,80 (19,17-36,11)	34	21,98 (14,97-31,08)
Com histórico de violência	67	33,69 (24,31-44,56)	29	15,26 (9,35-23,93)	28	12,60 (7,59-19,48)	70	38,67 (26,91-51,91)

IC95%: intervalo de 95% de confiança.

Fonte: elaboração própria.

A Tabela 4 apresenta as RC brutas e ajustadas da associação entre sintomas depressivos, consumo de álcool e as mudanças no *status* de perpetração da VPI nas pessoas idosas. Pessoas sem sintomas depressivos com histórico de violência familiar apresentaram mais do que o dobro de chance de continuar perpetrando VPI em comparação ao grupo de referência (RC = 2,19; IC95%: 1,20-4,00). A presença de sintomas depressivos sem histórico de violência foi associada com a maior chance para continuar perpetrando VPI (RC = 4,44; IC95%: 1,18-16,60). Entre aqueles com presença de sintomas depressivos e histórico de violência, as chances de passar a perpetrar a VPI foram de 4,93 (IC95%: 1,10-21,30) e de 8,09 (IC95%: 2,34-27,97) de continuar perpetrando. No que se refere ao consumo de álcool, observou-se associação significativa entre consumo elevado e passar a perpetrar VPI. A análise ajustada mostrou uma RC de 2,77 (IC95%: 1,12-6,84) para esse grupo, em comparação àqueles que nunca consumiram álcool (Tabela 4).

**Tabela 4:** Análise bruta e ajustada dos fatores associados à mudança do *status* da violência perpetrada nos idosos do estudo de coorte EpiFloripa Idoso. Florianópolis, Santa Catarina, Brasil, 2013-2014 e 2017-2019.

Variáveis	Passou a perpetrar VPI		Parou de perpetrar VPI		Continuou perpetrando VPI	
	RC (IC95%)	RC (IC95%)	RC (IC95%)	RC (IC95%)	RC (IC95%)	RC (IC95%)
	Bruta	Ajustada *	Bruta	Ajustada *	Bruta	Ajustada *
Sintomas depressivos						
Ausência de sintomas depressivos sem histórico de violência	1,00	1,00	1,00	1,00	1,00	1,00
Ausência de sintomas depressivos com histórico de violência	1,73 (0,85-3,49)	1,62 (0,79-3,30)	0,98 (0,49-1,95)	0,96 (0,48-1,93)	2,30 (1,27-4,16)	2,19 (1,20-4,00)
Presença de sintomas depressivos sem histórico de violência	2,47 (0,50-12,21)	2,45 (0,40-12,3)	3,55 (0,91-13,81)	3,10 (0,78-12,28)	4,23 (1,16-15,41)	4,44 (1,18-16,60)
Presença de sintomas depressivos com histórico de violência	4,92 (1,15-20,96)	4,93 (1,10-21,30)	0,57 (0,06-5,51)	0,52 (0,05-5,02)	7,94 (2,34-26,95)	8,09 (2,34-27,97)
Consumo de álcool						
Nunca	1,00	1,00	1,00	1,00	1,00	1,00
Moderado	1,53 (0,71-3,26)	1,76 (0,80-3,82)	0,84 (0,39-1,79)	0,96 (0,43-2,10)	0,86 (0,45-1,64)	0,86 (0,44-1,68)
Alto	2,36 (1,00-5,56)	2,77 (1,12-6,84)	0,97 (0,39-2,43)	1,20 (0,46-3,18)	1,89 (0,93-3,84)	1,98 (0,94-4,18)

IC95%: intervalo de 95% de confiança; RC: razão de chances; VPI: violência por parceiro íntimo.

* Análise ajustada para idade, escolaridade e renda.

Nota: grupo de referência - indivíduos que não perpetraram e nem passaram a perpetrar.

Fonte: elaboração própria.

Na análise bruta e ajustada da associação entre os sintomas depressivos e o uso de álcool com as mudanças no *status* de vitimização de VPI (Tabela 5) observou-se que os indivíduos com histórico de violência familiar, mesmo sem sintomas depressivos, apresentaram uma chance 93% maior de continuar sofrendo VPI, em comparação ao grupo de referência. As pessoas idosas com presença de sintomas depressivos sem histórico de violência têm uma chance 704% maior de continuar sofrendo VPI em comparação às sem suspeita de depressão e sem histórico de violência. Chama a atenção que aqueles com presença de sintomas depressivos e com histórico de violência exibiram chances ainda mais elevadas, com 840% mais chances de passar a sofrer e 920% mais chances de continuar sofrendo.

**Tabela 5:** Análise bruta e ajustada dos fatores associados à mudança do *status* da violência sofrida nos idosos do estudo de coorte EpiFloripa Idoso. Florianópolis, Santa Catarina, Brasil, 2013-2014 e 2017-2019.

Variáveis	Passou a sofrer VPI		Parou de sofrer VPI		Continuou sofrendo VPI	
	RC (IC95%)	RC (IC95%)	RC (IC95%)	RC (IC95%)	RC (IC95%)	RC (IC95%)
	Bruta	Ajustada *	Bruta	Ajustada *	Bruta	Ajustada *
Sintomas depressivos						
Ausência de sintomas depressivos sem histórico de violência	1,00	1,00	1,00	1,00	1,00	1,00
Ausência de sintomas depressivos com histórico de violência	1,31 (0,62-2,77)	1,27 (0,60-2,71)	0,82 (0,42-1,59)	0,82 (0,42-1,60)	2,01 (1,12-3,66)	1,93 (1,06-3,51)
Presença de sintomas depressivos sem histórico de violência	2,44 (0,37-16,01)	2,78 (0,40-19,05)	4,64 (1,11-19,45)	4,17 (0,95-18,32)	6,95 (1,72-28,06)	8,04 (1,91-33,81)
Presença de sintomas depressivos com histórico de violência	8,40 (1,82-38,62)	9,40 (1,94-45,47)	1,90 (0,35-10,21)	1,96 (0,35-10,90)	9,49 (2,38-37,75)	10,20 (2,48-41,94)
Consumo de álcool						
Nunca	1,00	1,00	1,00	1,00	1,00	1,00
Moderado	1,20 (0,52-2,75)	1,20 (0,49-2,91)	0,78 (0,38-1,61)	0,80 (0,36-1,75)	1,19 (0,63-2,25)	1,19 (0,61-2,32)
Alto	2,16 (0,90-5,21)	1,93 (0,75-4,97)	0,86 (0,36-2,04)	0,87 (0,34-2,22)	1,88 (0,93-3,80)	1,96 (0,93-4,14)

IC95%: intervalo de 95% de confiança; RC: razão de chances; VPI: violência por parceiro íntimo.

Fonte: elaboração própria.

Nota: grupo de referência - indivíduos que não sofriam e nem passaram a sofrer.

* Análise ajustada para idade, escolaridade e renda.

## Discussão

Destaca-se, como principais achados, que parte expressiva das pessoas idosas esteve envolvida em situações de VPI no intervalo entre as duas ondas da pesquisa, seja ao iniciar ou ao manter a condição de perpetradores e/ou vítimas. A maior parte dos indivíduos, no entanto, permaneceu sem alteração do *status*, o que sugere que as transições relativas à situação de VPI são processos complexos e possivelmente influenciados por múltiplos fatores. No que se refere à perpetração, os resultados demonstram que tanto os sintomas depressivos quanto o consumo de álcool estão associados, sendo que a presença concomitante de depressão e histórico de violência aumenta de forma mais significativa a chance de continuar ou começar a perpetrar VPI. O alto consumo de álcool também foi um fator associado ao início da perpetração. Quanto à vitimização, a presença de sintomas depressivos quando combinada com histórico de violência mostrou-se um forte preditor tanto para continuar quanto para começar a sofrer VPI. No entanto, chama a atenção que os sintomas depressivos sem o histórico de violência estiveram associados exclusivamente à manutenção da vitimização, não se observando relação com a sua incidência inicial.

Na China, pessoas do mesmo grupo etário que responderam ao mesmo questionário, CTS-1, apresentaram taxa de vitimização entre 0,4% e 36,1% no último ano ^43^. Resultados próximos também foram observados com populações idosas de um estudo multicêntrico, no qual a prevalência variou de 0,5% a 22,4% ^41^. Em relação às mudanças de *status* observadas entre as duas ondas da pesquisa, a maioria dos participantes se manteve no *status* da violência perpetrada (70,16%) e sofrida (68,1%). As barreiras à procura de ajuda em pessoas idosas, como a vergonha, a autoculpabilização, o medo de consequências negativas para si e para a família, e o medo da institucionalização já foram amplamente descritas na literatura ^44^ ^,^ ^45^ e podem dificultar a interrupção e o reconhecimento da violência, contribuindo para a não notificação dos casos. A dependência funcional também pode influenciar essa situação, ao transformar a relação conjugal em um arranjo de cuidado cuja ruptura tende a ser mais difícil. Estudos apontam que a fragilidade física, a necessidade de cuidado e a sobrecarga do cuidador podem contribuir para a manutenção ou início da VPI em idades avançadas ^46^.

Normas de gênero também influenciam a subnotificação. Em mulheres, especialmente em relacionamentos longos, a violência pode ser naturalizada ou interpretada como parte da dinâmica conjugal ^47^. Em homens, pode haver ocultação da vitimização por romper com ideais de masculinidade ^48^. Somam-se a isso as limitações na detecção e notificação, contribuindo para a persistente invisibilidade desse fenômeno ^13^.

Contudo, neste estudo, observou-se que 15,63% das pessoas idosas deixaram de perpetrar e 18,69% deixaram de sofrer VPI ao longo do período analisado entre as duas ondas, evidenciando que a interrupção da violência é possível. Embora poucas pesquisas tenham explorado fatores de proteção contra VPI nessa população, as evidências existentes elencam o apoio social, o comportamento de busca de ajuda e a disponibilidade de serviços comunitários como potenciais elementos que contribuem para a interrupção do ciclo de violência ^49^.

Por outro lado, também foi constatado que 14,19% dos participantes passaram a perpetrar e 13,19% passaram a sofrer VPI durante o intervalo do estudo. Um estudo americano identificou taxas de incidência inferiores, sendo 0,41% para abuso físico, 1,12% para abuso sexual e 1,62% para ameaças no último ano em mulheres acima de 55 anos na atenção primária à saúde (APS) ^50^. Em relação aos novos casos de violência, um estudo qualitativo identificou que o início recente da violência em casamentos de longa duração, assim como a vitimização em novos relacionamentos, estão frequentemente associados a condições de saúde física ou mental de um dos parceiros ^51^. Além disso, a literatura aponta uma ampla gama de fatores de risco associados à perpetração nesse grupo etário, incluindo condições econômicas desfavoráveis, pertencimento a minorias étnicas, comprometimento cognitivo ou físico, dependência do parceiro, estresse do cuidador, fatores culturais, uso de álcool, depressão e exposição pregressa à violência ^6^ ^,^ ^49^. Esses fatores refletem a interação entre as desigualdades estruturais e as condições de vulnerabilidade que se acumulam ao longo da vida e influenciam tanto o risco de violência quanto as possibilidades de reconhecimento e acesso a apoio ^52^. Na interseção entre envelhecimento e gênero, observa-se uma lacuna na atenção: os serviços e estratégias de rastreio estão concentrados em mulheres em idade reprodutiva, e, nas pessoas idosas, predomina o foco em violências associadas à vulnerabilidade, com menor reconhecimento daquelas perpetradas por parceiros íntimos ^53^. Nesse contexto, os serviços de saúde, especialmente na atenção primária, desempenham um papel central na identificação, manejo e coordenação do cuidado, embora enfrentem limitações estruturais, organizacionais e de capacitação profissional, o que pode comprometer a resposta ao fenômeno ^53^ ^,^ ^54^ ^,^ ^55^. Ainda, as intervenções, quando acontecem, têm efeitos diferentes conforme o contexto, o perfil das pessoas idosas e suas vulnerabilidades sociais ^56^.

Neste estudo, entre as pessoas idosas avaliadas, a presença de sintomas depressivos sem histórico de violência foi associada à chance para continuar perpetrando VPI (RC = 4,44; IC95%: 1,18-16,60). Esse achado converge com os resultados da metanálise conduzida com pessoas acima de 16 anos, em que a depressão foi associada a um aumento de 7,4% e 4,8% na proporção de perpetração de VPI física no ano anterior entre mulheres e homens, respectivamente ^57^.

Os dados apontam que a depressão pode estar associada à maior chance de perpetração de VPI. Quando combinada ao histórico de violência na infância, essa associação torna-se ainda mais expressiva, como visto no presente estudo, com uma chance 8,09 vezes maior de continuidade na perpetração (IC95%: 2,34-27,97). Embora sejam raros os estudos que analisam simultaneamente essas duas variáveis, pesquisas anteriores indicam um aumento do risco para as análises feitas separadamente. Há evidências de que homens provenientes de lares violentos são mais propensos a acreditar nos direitos dos maridos de controlar suas esposas e a serem física e sexualmente abusivos (RC = 3,82) ^58^. Além disso, uma metanálise verificou que crescer em um ambiente familiar violento está associado a uma maior probabilidade de se tornar perpetrador de VPI, sendo a associação mais forte entre homens (r = 0,25; IC95%: 0,23-0,28) do que entre mulheres (r = 0,19; IC95%: 0,14-0,24) ^59^.

Esses achados reforçam a concepção da perpetração da VPI como um fenômeno multifatorial, influenciado pela interação entre condições de saúde mental e experiências adversas vividas ao longo da vida. Além da depressão e do histórico de violência, outros fatores comportamentais, como o consumo de álcool, desempenham um papel relevante. Neste estudo, o alto consumo de álcool esteve associado de forma significativa apenas ao início da VPI, aumentando em 177% a chance entre os indivíduos quando comparado aos que nunca bebiam, mas sem impacto na continuidade da violência ao longo do tempo.

Corroborando essa perspectiva, Murphy et al. ^60^ evidenciaram, em estudo longitudinal com homens adultos e idosos com diagnóstico de alcoolismo, que o consumo de álcool precedia a maioria dos conflitos, violentos ou não, e que a quantidade ingerida era significativamente superior antes dos episódios de agressão física. Em contraste, Stuart et al. ^61^ não encontraram associação estatisticamente significativa entre o abuso de álcool pelo parceiro e o risco aumentado de VPI, ressaltando a heterogeneidade dos contextos estudados. Esses resultados ressaltam a necessidade de compreender a perpetração da VPI como um fenômeno dinâmico e multifatorial que é acentuado na velhice ^33^ ^,^ ^62^.

Além de ser importante estudar a perpetração da VPI, é fundamental considerar também os fatores associados à vitimização. Nesse sentido, os dados do presente estudo indicam que a presença de sintomas depressivos, mesmo na ausência de histórico de violência pregressa, está significativamente associada à continuidade da vitimização, com uma razão de chances de 8,04 (IC95%: 1,91-33,81). Esses resultados são consistentes com o estudo de Cations et al. ^19^, que acompanhou mulheres idosas na Austrália e encontrou que aquelas com histórico de VPI estavam com maior chance de desenvolver depressão ao longo do tempo (RC = 1,36; IC95%: 1,11-1,67). Homens acima de 55 anos também demonstraram impacto semelhante: aqueles que sofreram VPI apresentaram mais sintomas depressivos e índices mais baixos nas subescalas de saúde mental ^63^. Embora os contextos dos estudos sejam distintos, os resultados convergem ao indicar que a vitimização por VPI em pessoas idosas tem impacto significativo na saúde mental, principalmente das mulheres, aumentando o risco de transtornos depressivos e da continuidade da vitimização.

Ainda quando os sintomas depressivos estavam combinados com histórico de violência infantil, para os participantes deste estudo, as maiores RC foram observadas, estando significativamente associadas à continuidade da vitimização, com um aumento de aproximadamente 920% na chance (RC = 10,20; IC95%: 2,48-41,94), assim como ao início da vitimização, com um aumento de cerca de 840% na chance (RC = 9,40; IC95%: 1,94-45,47). Esses resultados são compatíveis com a pesquisa de McDonald & Thomas ^64^ com pessoas acima de 55 anos, que identificou a depressão e o histórico de abuso na infância como fortes fatores de risco para a vitimização futura. De forma semelhante, outro estudo com pessoas acima de 60 anos de descendência chinesa nos Estados Unidos verificou que indivíduos que relataram maus-tratos na infância apresentaram maior probabilidade de vivenciar VPI (RC = 2,57; IC95%: 1,78-3,71) e de relatar abuso quando idosos (RC = 5,53; IC95%: 4,01-7,64) ^65^.

Esses achados indicam uma forte inter-relação entre as experiências de violência familiar na infância, saúde mental e a vitimização por VPI, e embora analisadas isoladamente na literatura, ambas as variáveis têm sido consistentemente apontadas como fatores de risco para a violência em fases posteriores da vida. Em pessoas idosas, evidências mostram que o histórico de violência infantil está associado à depressão de início tardio ^28^ ^,^ ^66^, o que reflete a complexidade dessa relação.

Os resultados deste estudo reforçam a importância de aprofundar a compreensão dos mecanismos teóricos que articulam sintomas depressivos, consumo de álcool e VPI, uma vez que essas variáveis podem se relacionar por diferentes vias. Estudos prévios indicam que, em adultos e idosos, a depressão ou o sofrimento psicológico pode atuar como mediador na associação entre vitimização por VPI e uso de álcool ^67^ ^,^ ^68^ ^,^ ^69^ ^,^ ^70^. Em mulheres adultas, a vitimização por VPI foi identificada como mediadora na relação entre depressão e perpetração de VPI ^57^, e não foram observadas evidências consistentes de mediação pelo uso de álcool nesse contexto. Em conjunto, esses estudos sugerem que os mecanismos de mediação envolvendo depressão, álcool e VPI são complexos, contextuais e possivelmente dependentes de características populacionais, reforçando a importância de modelos analíticos que considerem as múltiplas vias explicativas.

Evidências longitudinais indicam uma relação bidirecional entre a VPI e a depressão em mulheres adultas, em estudos conduzidos em Ruanda ^71^ e na Ásia ^72^. Em idosos, estudos indicam que a exposição à VPI está associada ao desenvolvimento de sintomas depressivos ^21^ ^,^ ^73^. De forma complementar, há evidências de que a depressão pode aumentar o risco de VPI ^74^, sugerindo a plausibilidade de uma relação bidirecional entre esses fenômenos, ainda que raramente testada no mesmo conjunto amostral.

Na interpretação dos resultados, as limitações do estudo devem ser consideradas. A VPI foi mensurada usando-se o instrumento CTS-1, cujas propriedades psicométricas são reconhecidas na língua portuguesa, no entanto, não foi desenvolvido para populações idosas, o que pode reduzir a sensibilidade da medida. Por basear-se em autorrelato e na frequência de atos, e medir apenas violência física e psicológica, o instrumento não descreve o contexto da situação de violência. Apesar dessas limitações, o CTS-1 permanece como uma das medidas mais utilizadas em estudos populacionais sobre VPI ^75^. A violência pregressa também pode ter sido subestimada, pois depende da recordação de eventos ocorridos décadas antes da coleta de dados. Também por investigar violências em um período histórico no qual o castigo físico e a violência familiar eram mais tolerados socialmente e frequentemente compreendidos como formas de educação. Embora relatos retrospectivos de violência na infância possam estar sujeitos a viés de memória, estudos metodológicos ^76^ e evidências de coortes ^77^ populacionais indicam que esse viés tende a ter um impacto limitado nas associações estimadas e que tais medidas permanecem válidas e amplamente usadas em pesquisas epidemiológicas sobre violência ao longo do curso da vida.

Outra limitação está relacionada ao tamanho da amostra, o que dificulta a possibilidade de realizar algumas análises estratificadas por sexo e tipos de violência. Os intervalos de confiança amplos observados em algumas estimativas refletiram menor precisão estatística nas análises com subgrupos reduzidos e desfechos multinomiais. Achado esperado devido à baixa frequência de alguns eventos e à combinação de exposições. No entanto, as estimativas apresentaram consistência na direção e magnitude dos efeitos, sem evidência de instabilidade e coerentes com a literatura. Dessa forma, os intervalos amplos indicam uma limitação de precisão, sem comprometer a validade das associações.

Apesar dessas limitações, este estudo apresenta pontos fortes por ter sido realizado apenas com pessoas idosas e por identificar situações vivenciadas na infância combinadas com a presença de sintomas depressivos atuais e como estes podem influenciar a vitimização e a perpetração de VPI em estágios futuros da vida.

## Conclusão

Constatou-se que a VPI está presente de forma significativa nas relações entre pessoas idosas, tanto na perpetração quanto na vitimização, com prevalências semelhantes observadas entre homens e mulheres. A análise temporal indicou que a maioria dos participantes manteve o *status* de presença ou ausência de violência ao longo do tempo, embora mudanças tanto de interrupção quanto de início tenham sido registradas, permitindo melhor compreensão dos padrões de mudança. Este estudo é importante por explorar um tema ainda pouco abordado, focando na vitimização e na perpetração da violência dentro de relacionamentos íntimos em pessoas idosas.

Em relação ao principal achado do estudo, a presença de sintomas depressivos, sobretudo quando combinada ao histórico de violência na infância, esteve associada a um aumento das chances tanto de perpetração quanto de vitimização de VPI em pessoas idosas. O alto consumo de álcool esteve associado ao início da perpetração de VPI, mas não à sua continuidade e nem à sua vitimização, sugerindo um possível papel como fator precipitador, mais do que na manutenção do comportamento violento.

Do ponto de vista clínico e das políticas públicas, os resultados deste estudo reforçam a necessidade de formação de profissionais capacitados para o reconhecimento da VPI na população idosa, considerando a sua frequente coexistência com sintomas depressivos, uso de álcool e histórico de violência ao longo da vida. Profissionais que atuam na APS devem ser incentivados a incorporar o rastreamento sistemático de sinais de VPI e de sofrimento psíquico, mesmo quando estes não se apresentam de forma explícita, incluindo ações em saúde mental com o cuidado centrado na pessoa. O apoio à estruturação da rede de atenção à violência ou à articulação dentro dela no território é imprescindível para um cuidado contínuo e integrado, acionando os pontos intra e intersetoriais. A articulação com a rede de proteção dos direitos da pessoa idosa e a Rede de Atenção Psicossocial podem favorecer o fortalecimento de vínculos pessoais e comunitários.

Nesse contexto, destaca-se a importância de estratégias voltadas à prevenção intergeracional da violência, considerando as estratégias de fortalecimento de vínculo parental, os métodos de educação sem violência e o estímulo da comunicação não violenta entre o casal com abordagens integradas que considerem os fatores estruturais e culturais. A atenção à saúde mental, especialmente no que se refere à identificação e ao manejo de sintomas depressivos, configura-se como um componente central nas ações de prevenção e enfrentamento da VPI na população idosa. Nesse sentido, recomenda-se o desenvolvimento e a implantação de protocolos para a abordagem da VPI na APS, que considerem as especificidades da população idosa e as inter-relações entre diferentes gerações envolvidas nas dinâmicas de violência. Como direcionamento para pesquisas futuras, recomenda-se a realização de novos estudos longitudinais que forneçam dados mais detalhados sobre o momento e a intensidade da violência, a fim de compreender de maneira mais aprofundada os efeitos da violência doméstica desde a infância até o envelhecimento.

## Data Availability

Os dados de pesquisa estão disponíveis mediante solicitação à autora de correspondência.
